# Prediction and Analysis of Heart Failure Decompensation Events Based on Telemonitored Data and Artificial Intelligence Methods

**DOI:** 10.3390/jcdd10020048

**Published:** 2023-01-28

**Authors:** Jon Kerexeta, Nekane Larburu, Vanessa Escolar, Ainara Lozano-Bahamonde, Iván Macía, Andoni Beristain Iraola, Manuel Graña

**Affiliations:** 1 Vicomtech Foundation, Basque Research and Technology Alliance (BRTA), 20009 Donostia, Spain; 2 e-Health Department, Biodonostia Health Research Institute, Paseo Dr Begiristain s/n, 20014 San Sebastián, Spain; 3 Computational Intelligence Group, Computer Science Faculty, University of the Basque Country, UPV/EHU, 20018 San Sebastián, Spain; 4 Cardiology Department, School of Medicine, Basurto University Hospital, 48013 Bilbao, Spain

**Keywords:** machine learning, heart failure, decompensation, monitoring, XGBoost, logistic regression, supervised classification

## Abstract

Cardiovascular diseases are the leading cause of death globally, taking an estimated 17.9 million lives each year. Heart failure (HF) occurs when the heart is not able to pump enough blood to satisfy metabolic needs. People diagnosed with chronic HF may suffer from cardiac decompensation events (CDEs), which cause patients’ worsening. Being able to intervene before decompensation occurs is the major challenge addressed in this study. The aim of this study is to exploit available patient data to develop an artificial intelligence (AI) model capable of predicting the risk of CDEs timely and accurately. Materials and Methods: The vital variables of patients (n = 488) diagnosed with chronic heart failure were monitored between 2014 and 2022. Several supervised classification models were trained with these monitoring data to predict CDEs, using clinicians’ annotations as the gold standard. Feature extraction methods were applied to identify significant variables. Results: The XGBoost classifier achieved an AUC of 0.72 in the cross-validation process and 0.69 in the testing set. The most predictive physiological variables for CAE decompensations are weight gain, oxygen saturation in the final days, and heart rate. Additionally, the answers to questionnaires on wellbeing, orthopnoea, and ankles are strongly significant predictors.

## 1. Introduction

Heart failure (HF) is a life-threatening condition that affects the ability of the heart to maintain an adequate blood flow, and it is caused by changes in its structure or function; it typically has a poor prognosis. On many occasions, HF is a consequence of other diseases resulting in cardiac dysfunction. Some of its most common symptoms are shortness of breath, leg swelling, and excessive tiredness. These symptoms tend to worsen, leading to a reduced quality of life, increased dependency, and frequent hospitalizations.

Chronic HF disease mostly affects older adults. Its prevalence among people 75 and older is greater than 10% [1]. Moreover, for people over 65, HF is the leading cause of hospitalizations in developed countries. HF incidence is expected to rise due to population aging, chronic diseases, and the improved treatment for acute cardiovascular events. HF is a major economic burden for healthcare systems, accounting for 1–2% of the total healthcare expenditure in the EU and the USA [2]. Notably, the COVID-19 pandemic has accelerated these trends due to heart damage associated with infection [3], causing disruption to healthcare services, which has had a negative impact on prevention and efforts to delay progression.

HF may cause great suffering for families because most patients are highly dependent and require continuous care. Cardiac decompensation events (CDEs) may be triggered by an exacerbation of symptoms due to systemic or pulmonary congestion, leading to hypervolemia, which requires immediate treatment. This often results in hospitalizations [4], putting a high burden on the healthcare system, families, and caregivers. Additionally, rehospitalization rates after discharge are relatively high compared to those of other age-related diseases. Hence, avoiding CDEs via prediction and early intervention is a highly significant aspect of managing HF [5].

International guidelines [6] recommend that disease management programs focus on self-monitoring since it is crucial for preventing CDE- and HF-related hospitalizations. Home-based telemonitoring can help maintain the quality of care while reducing medical visits [7]. Telemonitoring usually requires a data collection of symptoms and physiological variables (temperature—T, heart rate—HR, blood pressure—BP, oxygen saturation—SPO2, etc.) by the patient or the caregivers. The challenge is to obtain insights from the data, ensuring accurate predictions on the basis of which better decisions can be made [8]. The emergence of artificial intelligence (AI) applications in clinical practice, the spread of mobile health (mHealth) resources, and novel non-invasive technologies monitoring vital signs, such as wearables, generate a growing interest among the medical community regarding providing evidence of their ability to improve care [9,10]. Although there are very limited data available on the effects of telemonitoring on HF patient care, several meta-analyses point to significant clinical benefits. Much of the focus is on the early detection of cardiac decompensations to prevent hospitalizations [11].

Most of the existing approaches to telemonitoring CDE prediction are based on rule systems triggering alarms when some variables are out of range [12]. This approach suffers from a high ratio of false positives and difficulties regarding scalability, preventing a wider adoption, as presented in [12]. Artificial intelligence (AI) models can model complex multivariate functions to provide accurate predictions based on training data. They can consistently stratify patients based on forecasted risk while enabling the scalability of solutions. 

Since 2014, the Hospital Universitario Basurto has implemented a monitoring program in which patients with HF are closely monitored in order to prevent cardiac decompensations or the worsening of their condition. For patients recruited in the program, vital signs are monitored, and the patient or the caregivers have to fill out an eight-question questionnaire. By applying a set of rules for alerts, clinicians can assess whether the patient status is worsening or not. If they detect that there is an increased risk of worsening, clinicians contact the patient to discuss their situation: hospitalization, treatment at home, or a possible false alarm.

In 2018, we reported the results of a computational study [12] in which various AI models were trained in the dataset collected in this program up to that date in order to predict CDEs. The computational study reported in this paper can be considered the second part of a previous study, in which the models have been trained with twice as many years’ worth of data. New computational approaches have been implemented, both in the data pre-processing pipeline and in the model training. The objective of this study is to apply these approaches (1) to improve predictive performance results, (2) to analyze the significance of recorded variables (by means of the feature importance extracted by an AI model) in order to learn about the pathophysiological evolution of HF decompensations, and (3) to create a predictive model with acquirable and applicable data that are easy to implement.

## 2. Materials and Methods

### 2.1. Dataset

The dataset used in the study was collected by clinicians at the Hospital Universitario Basurto (Osakidetza), Bilbao (Spain). The inclusion and exclusion criteria for the patient recruitment were the following: 

Inclusion criteria:

- Age of at least 18 years.
- Diagnosis of heart failure, as confirmed by a cardiologist.
- Recent CDEs that required diuretic adjustment (both oral and intravenous).
- Capable of using telemonitoring technology.

Exclusion criteria:

- Severe concomitant disease.
- Associated comorbidity with a life expectancy of less than 1 year.
- Dementia or moderate-to-severe cognitive impairment.
- Inability to use the required technology or a lack of disposition among relatives or caregivers to conduct the transmissions.
- Patients from the Bilbao–Basurto Health Organization area.

In total, 488 patients who met these inclusion/exclusion criteria were monitored from 2014 to 2022, with an average follow-up of 12.6 ± 9.6 (mean ± std) months per patient. This follow-up duration is similar to that in other studies [12]. These HF patients were older adults, aged 78 years on average, with a reasonable degree of independence, which is necessary for self-monitoring. Their average Barthel index was 83 [13]. The dataset construction was performed twice: in February 2018, providing data for the first published results [12], and in July 2022. Due to the procedures implemented to ensure patient data protection, the pseudonymized patient identifiers used in February 2018 were changed in 2022. Therefore, in the 2022 dataset, some patients may appear to be two different patients. Hence, the number of patients increases, while the lengths of the follow-up periods decrease. 

As shown in Table 1, the enrolled patients monitored five vital variables daily: weight, systolic and diastolic blood pressure (SBP and DBP), heart rate, and blood oxygen saturation. These variables were selected because they are widely recognized as indicators of patient well-being [8]. In particular, weight gain is an indication of fluid retention caused by cardiac decompensation in HF patients [14]. In 2018, the clinical professionals from the Hospital Universitaro Basurto considered that diuresis could be a significant variable, since retaining fluids is an indicator of worsening symptoms. For this reason, a diuresis decrease could be an early indicator of cardiac decompensation. Hence, it was included in the program as a daily variable.

In addition to vital signs, answers to eight questions on patient well-being were recorded. These questions are shown in detail in Table 2.

In our previous study [12], a filter was implemented to consider each monitoring day suitable for the study on the basis of alerts defined by the clinicians (e.g., days in which the patient had an oxygen saturation below 90). Therefore, not all monitoring days were used as data instances. In this study, all recorded monitoring days were used as instances to let the model itself find the necessary patterns.

The entire monitoring week was used as an input to predict the outcome, i.e., for each variable, seven columns were used (one column per day in each variable). Additionally, variables were replicated depending on how they were standardized:

- Normal standardization: the variables were standardized (by computing their Z-norm) considering the value of all patients in the training set. By applying this standardization, the trained AI model was expected to automatically find values that are indicators for decompensation (for example, a low oxygen saturation value).
- Week standardization: the variables were standardized using the values of the week per each patient, looking for patterns of increases/decreases or trends during the week. For example, weight gain is an indicator of decompensation, which would be reflected in this type of standardization. The variables resulting from this standardization were named as “trend” variables because it is expected that they represent the trend of the variable throughout the week.

In total, 133 variables were generated as follows. First, from the responses to the questionnaires, 56 variables were extracted (i.e., 7 days × 8 questions). Additionally, physiological variables—TAS, TAD, SO2, HR—and diuresis produced 35 variables (i.e., 7 days × 5 variables), which are subject to normal standardization. By adding weight to a set of physiological variables and applying week standardization to them, we obtained 42 additional variables (i.e., 7 days × 6 variables).

Regarding the nomenclature of these variables, their names comprise the tags of the variables, the numbered days, and the indicators of their standardization (the word “trend” if the variable is of the “week-standardization” type). For example, the diastolic blood pressure value of the previous day (1 day before) is named “DBP_1”. The count of days starts at 0, corresponding to the same day. The name of the variable corresponding to the weight six days before is named “Weight_trend_6”, and for the same day, it is named “Weight_trend_0”.

Some variables considered in the previous study [12] were discarded, in line with objective 3. Baseline data were discarded since they fail to represent any daily data and thus cannot be considered potential indicators of cardiac decompensation. Moreover, using baseline data may impede the transfer of the trained model to new cohorts or sites (since the new sites might not have measured them). Following the same line of reasoning, the alerts were also discarded, since they require the extra involvement of clinicians. Alternatively, it is preferable that the model directly learn from the data, without potential bias affecting clinicians’ expertise. Therefore, the model can be utilized in a more flexible way, using only the information from questionnaires and vital signs. 

The target variable of this study is the occurrence of CDEs suffered by patients in the near future. The current day was considered positive if the patient suffered a CDE in the following 7 days; otherwise, it was labeled as negative. During the first 4 years of follow-up, CDEs are categorized as “HF treatment at home”, “emergency room visits due to HF”, and “hospitalizations due to HF”. During the last 4 years, only “visits to the emergency room due to HF” and “hospitalizations due to HF” were considered. This limitation occurs because remote treatments are not documented in the second half of the program duration. Nevertheless, the neglected decompensations are less severe and rarer. In total, 3220 positive instances and 127,878 negative instances comprised the dataset. An extreme class imbalance poses a big problem when training classification models [15]. To perform the study with a balanced database, the negative class was randomly reduced to 4000 instances.

### 2.2. Model Training

#### 2.2.1. Train/Test Split

An initial split of 80/20 was made in the dataset to ensure that we evaluated the trained model using an unseen dataset (the testing set, corresponding to 20% of the original dataset) after training in the training set (80% of the original dataset). In order to not introduce bias in the results (more than one instance may exist for the same decompensation of a patient), the dataset split was conducted by patients. In other words, no patient in the training set also appeared in the testing set. Considering the high level of imbalance (many patients have no decompensation), there was the same proportion of patients with at least one decompensation in each set. Thus, the training set was not negatively affected by the random casuistry of having few events in the test dataset.

After the train/test data separation, we applied a cross-validation (CV) [16] method (10 folds) when training a model over the training set. The same process was followed when splitting the CV folds, i.e., the data of a patient are only found in one fold, and the folds are balanced in the same way as the training/testing datasets.

#### 2.2.2. Feature Selection: The Boruta Method

In the construction of the dataset, some variables were used twice (subjected to a different standardization) when they might not be indicators of decompensation. Therefore, these variables were discarded using a feature selection method known as the Boruta method [17]. This method creates random variables and compares their impact on the model (trained to predict the outcome). If any random variables had a greater impact than the real one, the usefulness of predicting the real variable was null. Therefore, the Boruta method is a very simple and effective method for discarding variables that do not contribute to the performance of the model.

#### 2.2.3. Missing Values

There are some variables that were not collected in some periods of the study (e.g., diuresis was only first recorded in 2018, and not for all the patients). Additionally, some variables were not reported on some days. All variables except for diuresis have a missing value rate lower than 2%. The diuresis missing value rate increased to 85% in the second data extraction, which could be considered a reason to discard this variable. However, after discussions with the clinicians, the variable was included in the study in case the model considers it to be highly predictive. Otherwise, it would be discarded during the feature selection using the Boruta method.

Missing values impede the classifier training computations; therefore, they were imputed using the KNN method [18]. This method looks for similar instances (always in the training fold) according to the rest of the variables in order to estimate the most likely missing value.

#### 2.2.4. Classifiers

Binary classification models are trained on a database and used to predict the outcomes from new data. In this study, different classifier models were evaluated in order to assess which one provides the best classification performance over the dataset. The library scikit learn [19] (in Python language [20]) was used to implement the following classifiers: Logistic regression [21], bagging classifier [22], XBBoost classifier [23], support vector machine [24], random forest [25], ExtraTrees classifier [26], AdaBoost classifier [27], and GradientBoosting classifier [28].

#### 2.2.5. Classification Performance Measures and Heuristic

Classification performance measures are used to determine the effectiveness of a classifier. In this study, the main performance measure used was area under the curve (AUC) [29]. In order to report the results, AUC values obtained in the different CV folds were plotted in boxplots, together with the achieved AUC values in the testing set. The boxplots were used to analyze whether the AUC values of different CV folds significantly differ from each other. Large differences between folds would indicate that it is not very certain how the model is going to behave with new, unseen data.

In addition, the training AUC value was calculated, i.e., the AUC obtained using the training dataset as the test data. The objective of computing this value was to determine if the model was overfitting the training data and see if it could learn general patterns (the lower this value, the better).

Test values were iteratively calculated in order to automatically optimize the model hyperparameters. A heuristic was used to consider which model is the best. This heuristic minimizes the difference between the train and test AUCs and maximizes the value of the test AUC (test of CV folds). Using this heuristic, hyperparameters were optimized, as discussed in the following section.

#### 2.2.6. Hyperparameter Tuning

Each classifier has some hyperparameters that can be tuned to provide better results in each database [30]. In order to find the best fit, a grid search over the space of values of the hyperparameters was performed. In addition, after the optimal value selection of each hyperparameter, a grid search was automatically repeated with similar hyperparameters to look for the best model. This greedy search is expected to find the best possible combination of hyperparameters for the predictive models.

## 3. Results

### 3.1. Feature Selection: The Boruta Method

By applying the Boruta method, the number of variables was reduced from the initial 133 variables to 42 variables. In other words, 91 variables were discarded because the Boruta method did not find them to be more significant than noise.

Regarding the trend variables (week standardization), the Boruta method recorded almost all weight variables from previous days. Oxygen saturation was maintained for three days of the previous week. The remaining variables were practically discarded.

Regarding variables with the usual normal standardization, the Boruta method recorded the oxygen saturation and heart rate variables for all days. For diastolic/systolic blood pressure, the Boruta method recorded variables on the final days. Finally, in the case of diuresis, the Boruta method practically discarded all variables from the previous day (this may be due to the limitations of the data on diuresis). Regarding the questionnaires, Boruta emphasized the last answers of “well-being”, “ankles”, and “walks” and considered the answer of all orthopnoea days. The complete relation of retained variables can be examined below in the article, where the logistic classifier feature importance is presented.

### 3.2. Cross-Validation

**Figure 1:**
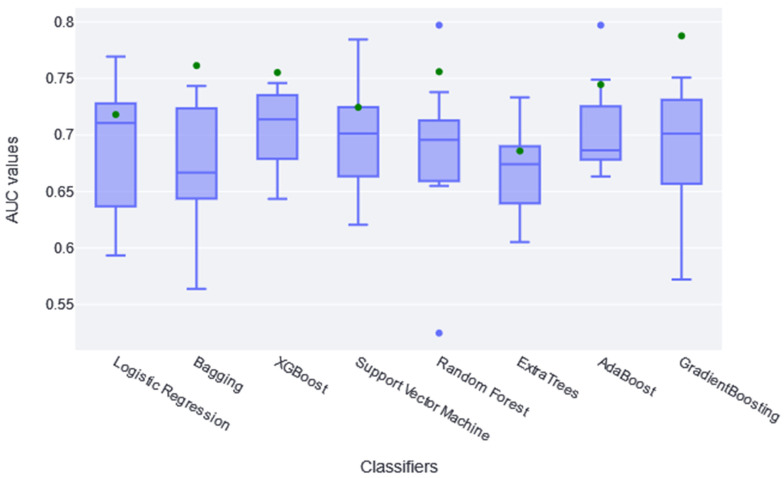
The boxplots in this figure represent the different AUC values obtained in each folder of the CV (10 AUC values per classifier; in blue). The green dot represents the AUC value obtained applying the model to the training set.

Once the number of variables was reduced by applying the Boruta method, the different classifiers were trained, and hyperparameter optimization was carried out. The performance results obtained for each CV fold are summarized in the boxplot of Figure 1. These boxplots make it possible to analyze the sparseness of the different AUCs, the median obtained, and even the outliers that may appear in any fold. The green dot represents the AUC value obtained in the same training set once the model has been trained in the whole set.

According to results shown in Figure 1, both LogisticRegression and GradientBoostingClassifier obtained diverse AUC values across the different folders; RandomForestClassifier and AdaBoostClassifier had the highest atypical AUC values, while the RandomForestClassifier AUC values are both above and below. The AUC value in the training set (green point) is within the boxplot in the logistic, SVC, and ExtraTree classifiers, indicating that it learned the general pattern without overfitting. However, almost all classifiers have similar train AUC values compared with CV AUCs. No significant differences were found between classifiers using the t-test statistic, except between XGBoost and ExtraTree (*p*-value of 0.04). Therefore, it is generally assumed that they all extracted the general pattern of the data in this sense. Considering these two aspects, together with the absolute AUC values, it was determined that XGBoost performed slightly better; therefore, it is considered the best fit for this study. It achieved a median AUC value of 0.72 in CV. Recent studies found that XGBoost achieves some of the best results in similar studies [31,32].

### 3.3. Testing Set

**Figure 2:**
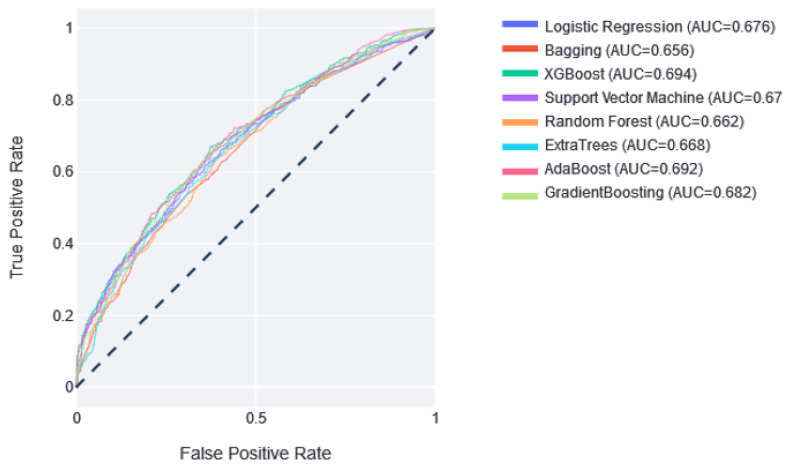
The ROC curves of the classifiers in the test partition. Next to them is the value of the area under this curve. Dotted line represents AUC value of 0.5, which means random prediction.

Once the best model was selected, the predictive ability of the models in the test set was analyzed. Figure 2 shows that the best result was obtained by XGBoost, with an AUC of 0.694, and the worst was obtained by Bagging, with an AUC of 0.656.

### 3.4. XGBoost

**Figure 3:**
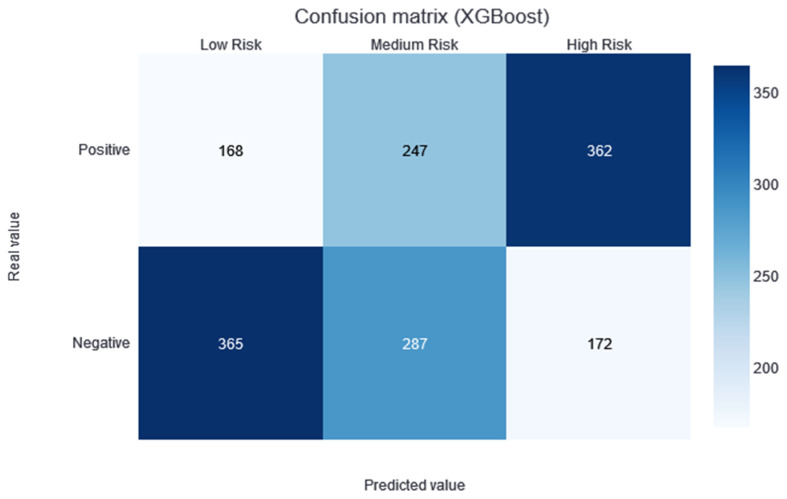
Confusion matrix of the XGBoost model results, divided into low-, medium-, and high-risk categories.

Two thresholds of risk were selected according to the results in the training set. Patients belonging to the training set were divided equally into three risk categories (obtaining two thresholds to obtain three groups): the interval from no risk to the first threshold value—low risk; the interval between the two thresholds—medium risk; and a final interval—high risk. In this way, although not guaranteed, the patients should be uniformly divided into the different risk categories. Different alerts are also created for these three slots: Low Risk, Medium Risk, and High Risk. As shown in Figure 3, the results of applying this methodology and partitioning the test data with the trained XGBboost classifier are shown. According to this confusion matrix, in the high-risk category, a precision of 0.67 is obtained. Moreover, the low-risk category is much more likely to be correctly predicted as negative. The medium-risk category maintains a balanced prevalence of both classes.

**Figure 4:**
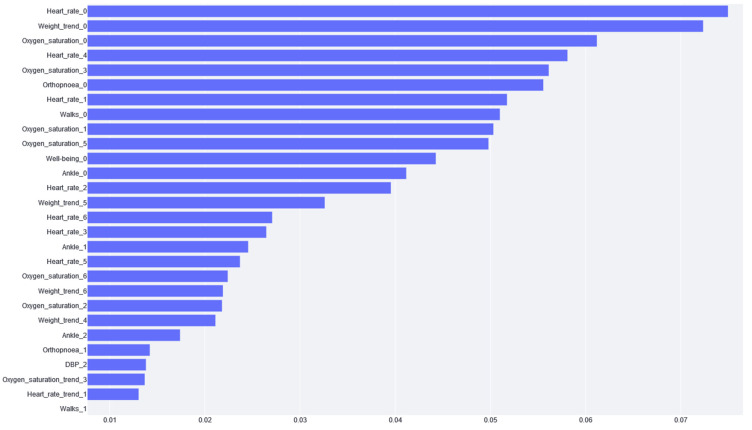
The importance given to each variable by the XGBoost model. The variable name is the acronym for the variable or the tag for the questionnaire. The number next to the name stands for the day of the variable value report (e.g., if it is zero, it is the variable value for the same day of risk prediction). Note: Variables with zero importance are not shown in the figure.

The feature importance of XGBoost is calculated from the prevalence of the variables along the trees in the ensemble, which is internally calculated during training. In other words, if a variable appears in many decision nodes, it gains importance, but it is impossible to know if this increases or decreases the risk. Figure 4 shows that the most significant variable according to XGBoost is the cardiac frequency on the last day, followed by weight gain.

### 3.5. Logistic Regression

Although the AUC values obtained in the cross-validation folders with logistic regression were very dispersed, the specific results of the logistic regression model were discussed for two reasons (in line with objective 2 of the study): (1) the results are very easy to interpret and clearly demonstrate what the model has learned; (2) you can study how each variable affects the feature importance, i.e., you can see which variables positively and negatively impact the performance of the classifier.

**Figure 5:**
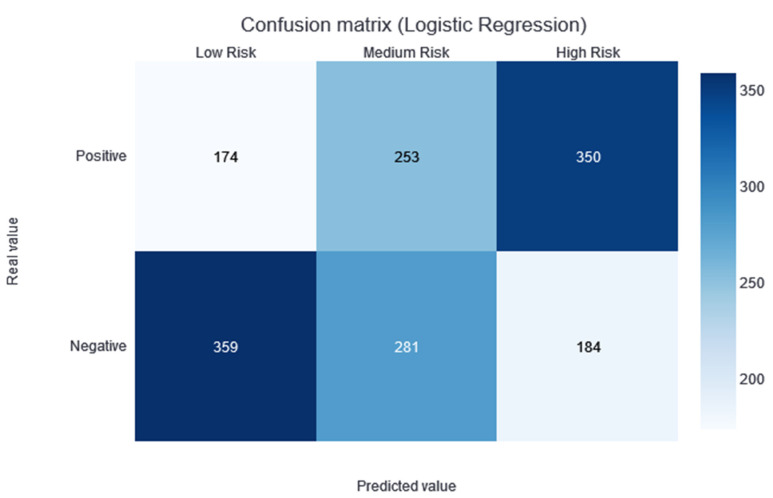
Confusion matrix of the logistic regression model results, divided into low-, medium-, and high-risk categories.

Figure 5 shows the confusion matrix for the results of the logistic regression, following the same process of separation into three categories, as previously described.

**Figure 6:**
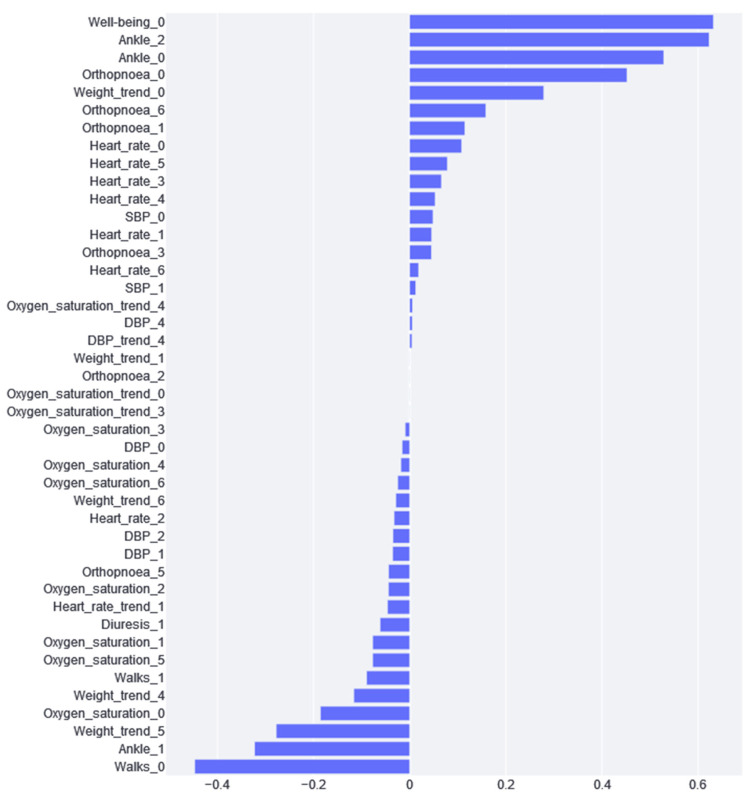
Feature importance calculated from the logistic regression model.

Figure 6 shows the feature importance results of the logistic regression model. From this figure, we can appreciate the impact of the different variables. If the impact is positive, the greater the variable and, thus, the greater the risk. Conversely, if the impact is negative, the higher the value of the variable and the lower the risk. Moreover, considering variables that are closely related to each other, we can draw different conclusions. 

As can be seen in Figure 6, the variable with the greatest impact is “well-being”, i.e., if the patient says that he/she feels worse, the probability that he/she is at a higher risk of cardiac decompensation. Although this variable has the greatest positive impact, we can see that weight is a very recurrent variable with a great impact. We found that weights 4, 5, and 6 have a negative impact, and weight 0 has a positive impact. This is because this variable is standardized according to the week values; therefore, if a patient experiences a negative impact from weights 4, 5, and 6 and the day 0 weight is positive, this means that weight gain is an indication of cardiac decompensation. On the other hand, the variables with the greatest impact are “Ankle_0” and “Ankle_2”. If the patient has been reporting for more than one day, this combination could mean that the patient has swelling ankles, and, thus, the risk of CDE is substantially increased. What does not fit in this interpretation is that the “Ankle_1” impact is negative.

## 4. Discussion and Conclusions

This study attempted to predict CDE based on monitored data in patients with chronic heart failure. It intends to improve the models previously presented in [12] with twice as much data (8 years instead of 4 years) and with new computational approaches in order to (1) see if better results are achieved, (2) study the impact of the variables, and (3) build a model that will be easy to deploy in new cohorts for their validation and usage.

The model providing the best performance results is XGBoost, with an AUC median value of 0.72 in the CV. No statistical difference was found during the t-test conducted against the rest of the classification models (except against the ExtraTree classifier; *p*-value of 0.04). However, taking into account the median AUC value, the dispersion of the AUC values (see the boxplots in Figure 1), and the AUC value over the training dataset (see the green point in Figure 1), XGBoost is considered to be the best model for this dataset. By making the three-category risk partition (see Figure 3), it can be seen that most of the positive instances of the testing set are well-classified in the high-risk category, achieving a precision of 0.67. 

This approach achieves more stable and better results than the previous study. Although the AUC value in the test dataset did not improve by much (from 0.65 to 0.69), the dispersion of the AUC values in the CV was much lower in this study than its predecessor. Therefore, despite a modest improvement, the results are more reliable, which is an important aspect of clinical practice. Moreover, these results must be considered with the limitations added to the database (in line with objective 3 of this study). First, some variables were removed, such as baseline data (age, how long ago the patient was diagnosed with the disease, some baseline questionnaires, etc.), as some variables may have caused problems regarding replicability and transfer to other sites. For example, there are variables that are not very commonly extracted (such as left ventricular ejection), and some local questionnaires are not used worldwide. Moreover, these variables are not very predictive since they do not represent the patient’s daily information. Second, customized patient alerts were discarded to avoid conflicts with the clinician’s expertise when transferring the patient to other locations. Finally, for the last four years of the study period, the use of home treatments was recorded; therefore, this missed information may mislead the training process. 

In the previous study, only the weight trend was used. In this study, the trend of the rest of the variables was analyzed to check if they might be a CDE predecessor. Unfortunately, the Boruta feature selection filter discards most of these variables. Therefore, we conclude that the trend is not significant in the rest of the variables, at least in the context of this study.

Once the models were trained, the impact of each variable on the prediction performance was studied. In the case of XGBoost, the most important variables are related to the last-day recordings of vital variables. Regarding the questionnaires, the most important variables are those concerning well-being, ankles, and orthopnoea. Patient self-assessments are also useful for predicting mortality and hospitalization. In the literature, strong correlations have been observed between the scores and functional class and the number of HF CDEs that require hospitalizations: worse self-assigned scores are associated with increased hospitalization rates, a worse quality of life, and decreased survival [33,34,35]. These variables are expected to be the indicators of HF cardiac decompensation because they indicate the worsening of the patient. Although this analysis can be conducted using the XGBoost model, logistic regression feature importance traditionally provides a much better comprehension of the model behavior. In this case, we found that weight gain is the most important variable (taking into account the importance that it gives to all weight variables), which is in agreement with the literature: Chaudhry et al. [14] found significant increases in body weight, beginning at least 1 week before hospitalization for HF. Moreover, during this time period, the risk of HF hospitalization increases in a monotonic fashion with increasing amounts of weight gain. Changes in weight, especially over short periods of time, can be good indicators of volemic worsening. However, many studies on this subject are controversial, indicating that little or no weight gain is observed before an episode of decompensation or that modest weight loss is observed after the clinical compensation of an acute HF CDE. In many cases, cardiac decompensation may occur, not due to the build-up of fluid but due to water redistribution from the periphery to the lungs via neurohumoral and inflammatory acute activation, leading to cardiac and vascular alterations that promote reduced venous capacitance and increased peripheral arterial resistance [36].

Moreover, the feature importance of logistic regression (see Figure 6) provides additional insights. The answers in the questionnaire regarding the final days of well-being, ankles, and orthopnoea provide relevant information about the increased risk of suffering from decompensation. On the contrary, we can appreciate that if a patient can go walking, as on previous days (Table 2, tag “Walks”), they are more likely to not experience decompensation. All of this information, which could seem trivial, is confirmed using a real data analysis. The clear explainability of the logistic regression model inclines clinicians to rely on this model and effectively implement it in their daily workflow.

On the contrary, logistic regression explainability has also made it easier to detect apparent contradictions. This is the case for the ankles’ variables. The values from the last day and two days before diagnosis have a risk-increasing impact, but the value for one day before has a decreasing risk impact. There is no clear explanation for this paradox; therefore, future work should be carried out to analyze whether this variable should be included in or removed from the study.

By comparing the feature importance from both XGBoost and logistic regression, “Heart_rate_0” appears to be highly significant in XGBoost (see Figure 4), but not so much in the logistic regression (see Figure 6). This could be explained by the inherent characteristic of the heart rate variable, where extreme high and low values are considered bad (in any patient, not specific to HF) and the middle values are adequate. XGBoost can handle this because it is based on trees and can thus differentiate between high and low values. In addition, because of this double split, this variable is used more often by the trees, and, hence, it has higher importance than its real impact. Logistic regression cannot handle these high and low values; therefore, it gives heart rate variables less importance because it cannot learn from them.

In addition, this model can be very helpful to clinicians for prioritizing patient care. Although it is not perfectly classified, it can attribute a risk value to each patient that can be used to carry out a triage. Given that clinicians closely review patients’ statuses and make regular calls if they notice that something is not right, this risk value can provide a way to prioritize patients who are deemed to be in the worst shape. This approach avoids the principal problem that predictive models have in clinic practice (the aim is to avoid, at all costs, the casuistry in which a patient is not attended to because their estimated risk is low); it only changes the attendance order of patients so that all patients will be attended to. 

Moreover, the models implemented in this study can be used to assess the impact of each variable in each prediction. This feature is very useful in the actual clinical practice because it allows the clinicians to perceive the reasoning for the high/low risk of a patient. This feature improves clinicians’ reliability in risk assessments; therefore, it is more likely that they will use it.

Lastly, this study attempted to develop an easy-to-use model in other hospitals/sites (Objective 3). Although this is a challenge that is not usually approached when analyzing predictive models, in this article, we emphasized it due to past issues that we faced when replicating the previous model [12] in other sites. In the ongoing European SHAPES project [37], validation and pilot studies of the previous model are being carried out in three pilot sites (three European hospitals). It was necessary to replicate and extract the data used in the previous model, which can be a great challenge depending on the variable and the site where it was being replicated. Even some variables, such as the alerts, were omitted. Therefore, one of the objectives of this study was to achieve the best classification results while always bearing in mind that the model was intended for use in real patients. To achieve this objective, only the necessary monitoring variables were used. In addition, using Boruta feature selection, some questionnaires were discarded, which facilitated their use, since the patients have to answer fewer questions daily.

In future research, we intend to improve the performance results using new models that learn from data temporality. There are several emerging approaches to the exploitation of clinical data with temporal components, such as recurrent neural networks [38] and, more recently, attention-based models (also known as transformers) [39]. These models can be trained using temporal data instead of using each day as a feature. Hence, the next step for improving the predictive capacity is to apply these methods to this dataset.

## Figures and Tables

**Table 1:** Daily monitored vital data.

Tag	Description
Weight	Body weight (kg)
SBP	Systolic blood pressure (mmHg)
DBP	Diastolic blood pressure (mmHg)
Heart_rate	Oxygen saturation (%)
Oxygen_saturation	Heart rate (bpm)
Diuresis	Urine quantity (mL)

**Table 2:** Questionnaire that patients answered daily; previously presented in [12].

Tag	Question	Possible Answer
Well-being	Compared with the previous 3 days, I feel:	B/W/S *
Medication	Is the medication affecting me well?	Yes/No
New medication	During the previous 3 days, did I take any medication without my clinicians’ prescription?	Yes/No
Diet and exercise	Am I following the diet and exercise recommendations provided by my clinician and nurse?	Yes/No
Ankle	In the last 3 days, my ankles are:	B/W/S *
Walks	Can I go walking like previous days?	Yes/No
Shortness of breath	Do I have fatigue or shortness of breath when I lay down in the bed?	Yes/No
Mucus	Do I notice that I start coughing up phlegm?	Yes/No

* B/W/S = better/worse/same.

## Data Availability

Data are unavailable due to privacy or ethical restrictions.
